# Anatomic Variation in Morphometry of Human Coracoid Process among Asian Population

**DOI:** 10.1155/2017/6307019

**Published:** 2017-04-06

**Authors:** Manal Fathi, Pike-See Cheah, Umar Ahmad, M. Nizlan Nasir, Aye Aye San, Ezamin Abdul Rahim, Paisal Hussin, Rozi Mahmud, Fauziah Othman

**Affiliations:** ^1^Department of Human Anatomy, Faculty of Medicine and Health Sciences, Universiti Putra Malaysia, 43400 Serdang, Selangor, Malaysia; ^2^Department of Human Anatomy, Faculty of Medicine and Health Sciences, Zawia University, Zawia, Libya; ^3^Genetics and Regenerative Medicine Research Center, Faculty of Medicine and Health Sciences, Universiti Putra Malaysia, 43400 Serdang, Selangor, Malaysia; ^4^Department of Anatomy, Faculty of Basic Medical Sciences, Bauchi State University, PMB 65, Gadau, Nigeria; ^5^Department of Orthopedics, Faculty of Medicine and Health Sciences, Universiti Putra Malaysia, 43400 Serdang, Selangor, Malaysia; ^6^Department of Human Anatomy, Faculty of Medicine and Health Sciences, UCSI University, 56000 Cheras, Kuala Lumpur, Malaysia; ^7^Department of Imaging, Faculty of Medicine and Health Sciences, Universiti Putra Malaysia, 43400 Serdang, Selangor, Malaysia

## Abstract

Ethnic origin plays an important role in bone morphometry. Studies examining the influence of coracoid process have focused primarily on adults and have not included people from diverse Asian ethnic backgrounds. Our goal was to explore ethnic differences in morphometry of coracoid among Asian population. We performed morphometric measurements of coracoid process on cadaveric shoulders and shoulder CT scans from 118 specimens. The cadaveric sample included Indian (46%), Chinese (27%), and Myanmarese (27%) subjects, while the CT scans sample included Chinese (67%) and Malay (33%) subjects. The morphometric measurements were performed using digital caliper and software developed at Golden Horses Health Sanctuary (GHHS). In the Indian cadaveric shoulders, the coracoid process is better developed than the other groups with the exception of the tip width of coracoid process. There are significant differences in almost all measurements (*P* < 0.05) between the ethnic groups. On the other hand, the morphometry of coracoid process from CT scans data is bigger in Chinese than Malay subjects when stratified by sex (*P* < 0.05). Moreover, in all morphometric measurements, the females had smaller measurements than males (*P* < 0.05). Understanding such differences is important in anatomy, forensic and biological identity, and orthopaedic and shoulder surgeries.

## 1. Introduction

Morphometric variations of the bone are clinically important. The scapula itself is a complex anatomic unit, so this draws the interest and attention of scientists and researchers to study the morphometry of the scapula [1, 2]. One of the most important clinical aspects of the scapula is the coracoid process [3], and its morphometry has been a study area of interest to many researchers [4–6]. The early researchers in coracoid process measurements have found differences, and reference values have been set for some races. Gumina et al. [3] reported that the morphometry of the coracoid process of the scapula shows differences in shape, length, and direction in an Italian population. Gallino et al. [7] also studied the length of the coracoid process in Egyptian skeletal collection and observed that the length of the coracoid process is extremely variable. Rios et al. [5] had also set standards for the white and black Americans. However, there is insufficient data regarding the morphometry of coracoid process across Asian ethnic groups. Most studies have been conducted on Caucasian population. Based on the cited literature above, the present study hypothesized that differences in ethnicity play an important role in morphometry of coracoid process in Asian population. The study aims to (1) determine the morphometric measurements of the coracoid process amongst the Asian population and (2) determine whether the Asian population have similar coracoid process morphometry in both cadaveric and CT scans data.

## 2. Materials and Methods

**Figure 1:**
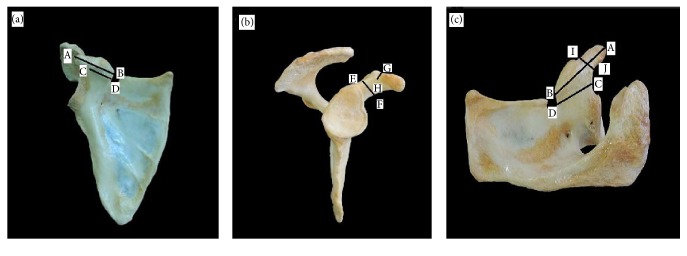
(a) Front view of the right scapula illustrated the measurement taken: (1) A-B line: the length of coracoid process (CP); (2) C-D line: the base width. (b) Lateral view of the right scapula illustrated the measurement taken: (1) E-F line: the base width of CP; (2) G-H line: the tip thickness of CP. (c) Superior view of the right scapula illustrated the measurement taken: (1) A-B line: the length of CP; (2) C-D line: the base width; (3) I-J line: the tip width of CP.

This research is an analytic cross-sectional study that was conducted on 118 specimens. It consists of two independent parts: an anatomical study of formalin-fixed cadaveric shoulders and a retrospective radiological study of computed tomography scan of scapulae. The work was approved by the Ethical Committee of the Universiti Putra Malaysia (UPM) under the code UPM/TNCPI/RMC/1.4.18.1 (JKEUPM)/F2, approval number FPSK_Nov (13) 24 (EXP). All subjects had given informed consent before computed tomography (CT) scans examinations were performed. The coracoid processes of all 118 scapulae were analysed. The following areas were measured from the coracoid process (Figure 1):

1. Length of coracoid process (distance from the tip to the end of horizontal part)
2. Tip thickness of coracoid process (superoinferior distance 1 cm posterior to the tip)
3. Tip width of coracoid process (anteroposterior distance 1 cm posterior to the tip)
4. Base height (maximum superoinferior distant of the base)
5. Base width (maximum medial-lateral distant of the base).

### 2.1. Anatomical Study of Formalin-Fixed Cadaveric Shoulders

**Figure 2:**
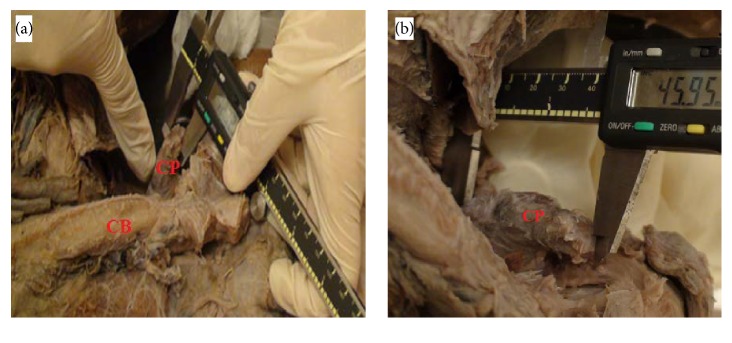
(a) Photograph showing digital caliper measuring the tip thickness of the coracoid process. (b) Photograph showing digital caliper measuring the length of the coracoid process.

The anterior aspect of 52 embalmed adult male shoulders was dissected. The age and race of the donors were recognized. In all cadaveric shoulders, the skin was separated from the deltoid and trapezius, and the deltopectoral groove was located. Next, the deltoid muscles were incised vertically to expose the coracoid process with all attachments. All attached muscles and ligaments were dissected out. After all attachments were dissected from their insertion, the anatomic feature of the coracoid process was visualized [8]. The measurements were carried out with digital caliper accurate to 0.01 mm (Mitutoyo 0- to 6-inch 150 models 500-321; Mitutoyo America, Aurora, IL, USA) (Figure 2). All anatomical investigations were performed in the Anatomy Dissection Hall of the Universiti Putra Malaysia. To normalize the measurements, only one investigator using the same instruments carried out all the measurements, which were done twice and averaged out. The exclusion criterion was the presence of any morphological changes affecting the normal anatomy of the coracoid process due to surgery, or shoulder trauma. In addition, the inclusion criteria were all cadavers with intact shoulders that had not undergone shoulder surgery and had not sustained any shoulder damage.

### 2.2. Radiological Study

**Figure 3:**
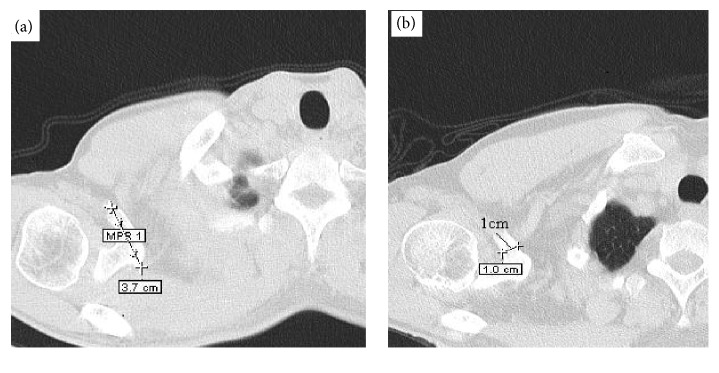
Axial CT scan images showing the measurement of the tip of the coracoid process.

**Figure 4:**
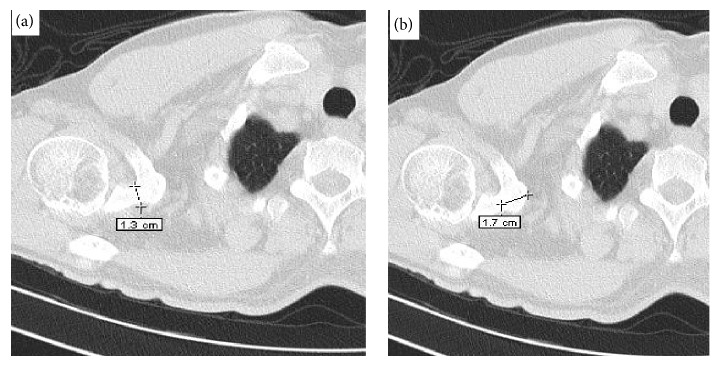
Axial CT scan images showing the measurement of the base of the coracoid process.

Retrospective study analysis of 66 CT scans of shoulders taken as part of a standard CT using chest protocol was performed. The CT images were acquired using a Siemens Somatom Emotion 6 (kV (kilo voltage) = 110, mA (milliampere) = 21, rotation time = 0.8 second, and slice thickness 1.3 mm). CT scan images were uploaded in DICOM format to medical imaging software (e-film, version 2.1.2, Merge Healthcare, Milwaukee, WI). Three sequential axial images (1.3-mm in thickness, original magnification) of each of the 66 sites were selected (*n* = 198) on each side using e-film (software application in an IBM-compatible PC). After the coracoid process with all anatomical landmarks on the scapula was acquired, the same anatomical landmarks were used across all the measurements by single investigator to ensure the accuracy of the measurements (Figures 3 and 4).

### 2.3. Statistical Analysis

The results were presented as mean ± standard deviation (SD) from dual studies that were performed independently. The normality of data distribution was checked by skewness and kurtosis level. The statistical analysis was carried out using one-way analysis of variance (ANOVA) for data from cadaveric study and differences between ethnic groups were separated using Tukey HSD post hoc test, while the data obtained from CT scan was analysed by independent *t-*test to determine the differences between the two ethnic groups. The gender differences, as well as the side differences, were evaluated separately for each measurement by independent *t-*test. The results are considered statistically significant when *P* values are less than 0.05 at a confident interval of 95%. Statistical Package for Social Sciences (SPSS) software, version 21, was used only for the statistical analysis.

## 3. Results

### 3.1. Anatomical Study of Formalin-Fixed Cadaveric Shoulders

#### 3.1.1. Study Subjects

A total of 52 male cadaveric shoulders (26 cadavers) were selected in this study. An expert anatomist did the selection of these cadavers based on their physical examination.

#### 3.1.2. Sociodemographic Characteristics of the Subjects

Table 1 provides the sociodemographic characteristics of the cadavers contained in the present study. Cadavers of different ethnic groups comprising Chinese, Indians, and Myanmarese were investigated in this study. The majority of the total cadaveric group were Indians (46%), followed by the Chinese (27%) and the Myanmarese (27%) as the least case. The age range for the cadaveric subjects was between 27 years and 62 years at the time of death with mean values of 45.38 ± 11.50 years. However, the majority of the cadavers were aged at the time of death between 30 and 62 years with a cumulative percentage of 88.4%.

#### 3.1.3. Coracoid Process Measurements

*Length of the Coracoid Process (LCP)*. Myanmarese subjects had the numerically smallest mean of the LCP (39.19 ± 1.38 mm) and the Indian subjects had the numerically largest mean LCP (43.32 ± 1.54 mm). The independent ANOVA test results between groups yielded statistically significant difference: *F* (2, 49) = 40.68, *P* = 0.001, *η*^2^ = 0.62. To evaluate the nature of the difference between the three means, ANOVA test was followed with Tukey HSD post hoc multiple comparison tests. Differences in the LCP among the three ethnic groups were observed, with Myanmarese subjects having the shortest coracoid process compared to both the Chinese and Indian subjects significantly (*P* < 0.001). However, there were no significant differences in LCP between the Indian and Chinese subjects (*P* > 0.05) (Table 2).

*Tip Thickness of the Coracoid Process (TTCP)*. Likewise, in the LCP, the Myanmarese subjects had the numerically smallest mean of TTCP (8.58 ± 1.03 mm) and the Indian subjects had the numerically largest mean of TTCP (11.47 ± 0.62 mm). The ANOVA was significant; *F* (2, 49) = 82.09; *P* = 0.001; *η*^2^ = 0.77. Follow-up tests were conducted to evaluate pairwise differences among the means, and there was a significant difference in TTCP between the ethnic groups. The Myanmarese and Chinese subjects had significantly smaller measurements than the Indian subjects (*P* < 0.001). In contrast, there was no significant difference between Myanmarese and Chinese subjects (*P* > 0.05) (Table 2).

*Tip Width of the Coracoid Process (TWCP)*. As shown in Table 2, there are no numerical differences in the mean TWCP among the three ethnic groups (Myanmarese subjects: 13.02 ± 1.32 mm, Chinese subjects: 13.17 ± 0.51 mm, and Indian subjects 13.63 ± 1.09 mm). The analysis of variance was carried out to assess the differences between the groups. There were no statistically significant differences between group means as determined by one-way ANOVA (*F* (2, 49) = 2.36, *P* = 0.104, *η*^2^ = 0.69).

*Base Height of the Coracoid Process (BHCP)*. Differences in the mean of BHCP among the ethnic groups were observed, with Myanmarese subjects having the numerically smallest mean (14.79 ± 0.88 mm) compared to the Chinese subjects and Indian subjects (15.26 ± 1.18 mm and 15.94 ± 1.33 mm, resp.). There is a significant difference between the ethnic groups in the result of one-way ANOVA test (*F* (2, 49) = 4.37, *P* = 0.01, *η*^2^ = 0.15). Despite the numerical difference between the Chinese and Myanmarese subjects, the Tukey HSD post hoc multiple comparison tests showed no significant difference between these two groups. On the other hand, there is a significant difference between the Myanmarese and Indian subjects with the Myanmarese subjects having the smallest mean (*P* > 0.05) (Table 2).

*Base Width of the Coracoid Process (BWCP)*. Similarly, the Myanmarese subjects had the numerically smallest mean (22.82 ± 0.78 mm), while the Indian subjects had the largest mean (*M* = 25.48 ± 1.49 mm). One-way ANOVA results (Table 1) showed there was a significant difference between the three ethnic groups (*F* (2, 49) = 4.37, *P* = 0.01, *η*^2^ = 0.15). Post hoc multiple comparison tests were conducted to evaluate pairwise differences among the means, and there was a significant difference in BWCP between the ethnic groups. The Myanmarese subjects had the significantly smallest mean (*P* < 0.001) followed by the Chinese (*P* > 0.05) and the Indian (*P* > 0.05) subjects (Table 2).

*Side Differences in Morphometry of the Coracoid Process*. An independent* t*-test was performed to test the hypothesis that the right and left coracoid process had a statistically significant difference in all measurements. The normality of data was checked, and data from both sides were sufficiently normal for the purpose of conducting a* t-*test (i.e., skewness < 2.0 and kurtosis < 9.0). The independent sample* t-*test showed nonsignificant difference between right and left coracoid process in all ethnic groups (*P* > 0.05) (Table 3).

### 3.2. Radiological Study

#### 3.2.1. Study Subjects

An expert radiologist selected a total of 66 shoulders' CT scans from 33 normal subjects. Those subjects were visiting Golden Horses Health Sanctuary (GHHS) for regular medical checkup.

#### 3.2.2. Sociodemographic Characteristics of the Subjects

Table 4 presents the sociodemographic characteristics of the subjects. The study group comprised 22 Chinese subjects (67%) and 11 Malay subjects (33%) with mean ages of 40.54 ± 4.22 years and 38.18 ± 6.80 years, respectively. The mean height, weight, and BMI of the Chinese subjects were 163.95 ± 9.40 cm, 62.27 ± 15.92 kg, and 23.17 ± 4.02 kg/m^2^, respectively, while for Malay subjects the average height, weight, and BMI were 166.45 ± 8.18 cm, 71.65 ± 13.73 kg, and 25.45 ± 4.13 kg/m^2^, respectively. Overall, the Malay subjects were heavier and taller and had a higher BMI compared to the Chinese subjects.

#### 3.2.3. Coracoid Process Measurements

*Length of the Coracoid Process (LCP)*. Differences in the LCP among the ethnic groups were observed numerically, with Chinese subjects having the larger mean (38.75 ± 3.32 mm) compared to Malay subjects (37.36 ± 2.68 mm). However, these differences are not statistically significant (*P* > 0.05) among all groups. Chinese males had a greater LCP (41.80 ± 1.47 mm) in comparison with their Malay counterparts (39.14 ± 1.16 mm). Similarly, Chinese females had a greater LCP (36.20 ± 2.02 mm) than Malay group females (34.25 ± 1.28 mm). Independent* t*-test results showed that these ethnic differences among males and females groups were statistically significant:* t* (32) = 5.62, *P* = 0.001,* t* (30) = 2.55, and *P* = 0.01, respectively (Table 5).

*Tip Thickness of Coracoid Process (TTCP)*. Ethnic specific variation in the mean TTCP was documented numerically. Malay subjects had TTCP mean of 9.45 ± 1.37 mm; by comparison, Chinese subjects had thicker tip (10.13 ± 1.39 mm). Independent* t-*test results showed these ethnic differences were not statistically significant (*P* > 0.05 among all groups). When ethnic groups were stratified by sex, among males, the tip of coracoid process was thicker in the Chinese subjects. These differences were statistically significant (*t* (32) = 2.70, *P* = 0.01). It was also thicker in females of the Chinese subjects (9.25 ± 0.94 mm) than Malay subjects (8.00 ± 0.75 mm). Similarly, the differences were statistically significant (*t* (30) = 3.38, *P* = 0.002) (Table 6).

*Base Height of the Coracoid Process (BHCP)*. Minor but significant ethnic variability was found for the coracoid process base height. Chinese subjects had the higher base of the coracoid process (14.90 ± 1.52 mm) whereas Malay subjects had marginally shorter bases (13.77 ± 1.87 mm). This similarity occurred across both sexes in all groups. When stratified by sex, Chinese females had slightly higher base (13.83 ± 1.52 mm) than their Malay counterparts (11.75 ± 1.28 mm) (*t *(30) = 4.60, *P* < 0.001). In males, the base height of the coracoid process was higher in Chinese subjects (16.20 ± 0.83 mm) compared to the Malay subjects who had shorter base (14.92 ± 0.91 mm) (*t *(32) = 4.20, *P* < 0.001) (Table 7).

*Base Thickness of the Coracoid Process (BTCP)*. The coracoid process base thickness was marginally greater in Chinese subjects than Malay subjects and these ethnic differences were not statistically significant (10.45 ± 2.06 mm, 10.36 ± 1.70 mm, resp.) (*P* = 0.85). When ethnic groups were stratified by sex, this similarity occurred across all the sexes and both ethnic groups showed no significant differences (*P* = 0.33 and *P* = 0.36, for males and females, resp.) (Table 8).

*Gender Differences in Morphometric Measurements of the Coracoid Process*. In order to test variations in morphometry of coracoid process between males and females, independent *t*-test was conducted. In the Chinese subjects, the length of the coracoid process in males (41.80 ± 1.47 mm) was reported to be significantly higher than females (36.20 ± 2.02 mm) (*P* < 0.001). In addition, the males had thicker coracoid process (11.20 ± 1.05 mm) than females (9.25 ± 0.94 mm) (*P* < 0.001). Among the base of coracoid process, the same results were reported with the males having a higher base (16.20 ± 0.83 mm) than the females (13.83 ± 1.04 mm) (*P* < 0.001). Also the males' coracoid bases were thicker (11.80 ± 1.60 mm) than the females' (9.33 ± 1.71 mm) (*P* < 0.001).

On the other hand, similar results were found in the Malay subjects with the males having longer and thicker coracoid process (39.14 ± 1.16 mm and 10.28 ± 0.82 mm, resp.) than the females' coracoid process (34.25 ± 1.28 mm and 8.00 ± 0.75 mm, resp.). Regarding the base of coracoid process, the males also had higher and thicker coracoid base (14.92 ± 0.91 mm and 11.28 ± 1.32 mm, resp.) than the females' coracoid base (11.75 ± 1.28 mm and 8.75 ± 0.88 mm, resp.). Overall, the males had bigger coracoid process than females in both ethnic groups (Table 9).

*Side Differences in Morphometric Measurements of the Coracoid Process*. In order to test the variations in morphometric measurements between right and left coracoid process, an independent sample *t*-test was conducted. This test found statistically nonsignificant differences in all ethnic groups' measurements (*P* > 0.05) (Table 10).

## 4. Discussion and Conclusion

Numerous reports have shown that the skeletal morphometry is influenced by different factors such as race and sex [9, 10]. The morphometry of coracoid process had previously been studied as a key structure and potential mediator in shoulder surgery and pathology [11]. The majority of these studies have been carried out on dry osteology [1, 3–7, 12–14], while others were done on cadavers [15–21]. In addition, few authors have performed morphometric analysis on in vivo populations using CT scans [11, 22, 23]. The large part of previously mentioned studies was done in different western populations. Although there have been many previous reports demonstrating that Asians have smaller bones than those of the western population [24], only two studies were carried out in Asian population (i.e., Thai and Indian) to study the morphometry of coracoid process [12, 14]. None of these were performed in Malaysia. Moreover, data comparing the differences within the various Asian nations seem to be lacking. The current study determined differences in the morphometry of the coracoid process between different Asian ethnic groups in both cadavers and CT scans.

### 4.1. Cadaveric Study

 The comparison of the results of the morphometric analysis obtained from the present cadaveric study with the results of other studies is as follows: the mean length of males' coracoid process in all three ethnic groups ranged between 39.19 ± 1.38 mm and 43.32 ± 1.54 mm and was smaller than Americans' (45.6 ± 4.2 mm and 46.3 ± 3.3 mm) (Dolan et al. [19]; Rios et al. [5], resp.), Germans' (46 ± 1.9 mm) [17], Polish's (44.6 ± 4.46 mm) [1], and South Africans' (44.5 ± 3.8 mm) [4]. On the other hand, these results were approximately similar to the results of the studies in [12] and [14] in Indian (41.0 ± 3.9 mm) and Thai (43.2 ± 3.5 mm) population, respectively. However, the existing results were similar to Terra et al.'s [21] in Brazilian and Galleno et al.'s [7] in Egyptian population (42.6 ± 2.6 mm and 41.1 ± 4.6 mm) and the measurements of our analysis were larger than Gumina et al.'s [3] in an Italian population. A possible explanation for this might be a mixture of sexes in the sample, as the sexes were not taken into account in previous studies. Regarding the tip thickness of CP in all male ethnic groups, it ranged between 8.58 ± 1.03 mm and 11.47 ± 0.62 mm and was thinner than those of the Brazilian (14.9 ± 1.2 mm) (Terra et al. [21]) and the American (13.5 ± 1.6 mm, Dolan et al. [19]) population. In contrast, it was thicker than the Egyptians' (7.19 ± 1.04 mm, Gumina et al. [3]). Gumina et al.'s result is likely to be related to a mixture of sexes in the sample or the measurements were taken on dry osteology. However, the present results were similar to the results from some different population such as German (9.6 ± 0.9 mm, Salzmann et al. [17]), Brazilian (8.37 ± 0.93 mm, Bueno et al. [6]), South African (9.0 ± 1.4 mm, Bhatia et al. [4]), and Thai (8.2 ± 0.9 mm, Piyawinijwong et al. [12]) population. The present results are more consistent with those of Piyawinijwong et al. [12] which is unsurprising because the tip thickness measurements by the rest of the authors were made at the thinner and distal coracoid tip, as opposed to measuring thickness at the middle of coracoid tip, where the coracoid is thicker and more important clinically [21]. In the present study, the mean tip width of the coracoid process in all ethnic groups ranged between 13.02 ± 1.32 mm and 13.63 ± 1.09 mm; these values compared to earlier works in other populations showed marked racial variation. The reported mean in Americans was 15.9 ± 2.2 mm and 18.3 ± mm 1.8 (Lo et al. [15], Dolan et al. [19] resp.), in Germans 15.2 ± 1.5 mm, and in South Africans 15.1 ± 1.6 mm (Bhatia et al. [4]). These studies presented broader coracoid tip than our results. The finding regarding the base heights of coracoid process in all ethnic groups ranged between 14.79 ± 0.88 mm and 15.94 ± 1.33 mm and the base of coracoid process in the currant study is shorter than South African [4], similar to German [17], and higher than American [5] population. On the other hand, the base width of coracoid process in this study ranged from 22.82 ± 0.78 mm to 25.48 ± 1.45 mm, which is wider than Germans' [17], thinner than South Africans' [4], and the same as Americans' [5]. This inconsistency may be because these studies measured the height and the width from different defined points or the point was undefined. The dimensions of the tip of the coracoid process were comparable to the values mentioned in literature [12]. However, the majority of the measurements of the base of the coracoid process could not be compared. This may be attributed to the lack of a precise definition of the base of the coracoid process in earlier studies [4]. The most interesting finding was that there were significant racial differences in all coracoid process measurements except the tip width of coracoid process. Conversely, no significant difference between black and white Americans was detected by Rios et al. [5]. The conflicting results of this study may be explained by variation in the genetic, environmental, and nutritional factors. Overall, there were no significant differences with regard to all distances related to the coracoid process when comparisons are made between the right and left sides. These results are similar to studies' results by Rios et al. [5], Bhatia et al. [4], Salzmann et al. [17], and Piyawinijwong et al. [12].

### 4.2. Radiological Study

In reviewing the literature, very little data was found on morphometry of the coracoid process on in vivo population using CT scans [11, 22, 23]. To the best of our knowledge, this is the first in vivo study in different Asian populations. The values of the length of the coracoid process in this computed tomographic study compared to previous studies of other populations showed obvious racial variation. The current study found that the mean coracoid length ranged between 34.25 ± 1.28 mm and 41.80 ± 1.47 mm. For male shoulders in all ethnic groups, mean coracoid length ranged from 39.14 ± 1.16 mm to 41.80 ± 1.47 mm. This length was smaller than those of the American males (45.0 ± 3.8 mm) [23]. The female coracoid length in all ethnic groups ranged between 34.25 ± 1.28 mm and 36.20 ± 2.02 mm which is also smaller than American females (41.5 ± 2.5 mm) [23]. In addition, Armitage et al. [22] conducted a computed tomographic assessment of the coracoid process on 23 Canadian shoulders; they reported a mean length of 16.8 ± 2.5 mm. Although the previous study was conducted in western population, their length values are shorter than ours, and the length measurements of the coracoid differ because in the present study the measurements were done along the longer outer surface length to the suprascapular notch whereas they measured the smooth undersurface length. Concerning the tip thickness of coracoid process, the values of the male's coracoid process ranged between 10.28 ± 0.82 mm and 11.2 ± 1.05 mm. Concerning the equivalent measurement for the female coracoid process of all the ethnic groups, the means ranged between 8.00 ± 0.75 mm and 9.25 ± 0.94 mm. In Canadian population, Armitage et al. [22] reported a mean tip thickness of coracoid process of 10.5 ± 1.7 mm from measurements obtained from CT scans data, which is very similar to our male values and higher than the female ones. These aspects can be attributed to the sex mixing, as the results were not stratified by sex. To the best of our knowledge, no published data are in place for comparison of the coracoid base height and thickness. When separating by race, this study reported significant differences between the Malay and the Chinese in all measurements except the base thickness in both sexes. These results corroborate the ideas of Hussain et al. [25], who suggested that there were significant differences in bone morphometry between the Malay and the Chinese. In addition, Tang et al. [26] documented statistically significant differences in the femoral head sizes in the previous mentioned ethnic groups. The present study has several limitations: firstly, being a retrospective study, secondly, the small numbers of subjects, thirdly, the mismatch in patient race distribution, and, finally, the shortage of female cadavers.

In conclusion, variations in morphometric measurements of coracoid process between different Asian ethnic groups were observed in both cadaveric and radiological studies. These findings are very important, which gave the clue that Asians coracoid process sizes are entirely different and cannot be generally applied throughout the Asian population. Furthermore, the results showed that the measurements of the coracoid process in Asian are smaller than those of western populations.

## Figures and Tables

**Table 1:** Distribution of the cadavers according to sociodemographic characteristics.

Variable	*n*	%
*Ethnicity*		
Indian	12	46
Chinese	7	27
Myanmarese	7	27
*Total*	26	100
*Gender*		
Male	26	100
*Age group (years)*		
20–30	2	7.6
30–40	7	26.9
40–50	7	26.9
50–62	9	34.6

**Table 2:** Morphometric measurements of the coracoid process of the scapula among Chinese, Indian, and Myanmarese subjects.

Measurements	Indian (*n* = 24)	Chinese (*n* = 14)	Myanmarese (*n* = 14)
Length of coracoid process (mm)	43.32 ± 1.54^a^	42.47 ± 1.02^a^	39.19 ± 1.38^b^
Tip thickness of coracoid process (mm)	11.47 ± 0.62^a^	9.08 ± 0.58^b^	8.58 ± 1.03^b^
Tip width of coracoid process (mm)	13.63 ± 1.09	13.17 ± 0.51	13.02 ± 1.32
Base height of coracoid process (mm)	15.94 ± 1.33^a^	15.26 ± 1.18^ab^	14.79 ± 0.88^b^
Base width of coracoid process (mm)	25.48 ± 1.49^a^	23.90 ± 0.76^b^	22.82 ± 0.78^c^

^abc^Values in the same row with different superscripts are significantly different at *P* < 0.05 based on one-way ANOVA and Tukey HSD post hoc test. Data were presented as mean ± SD.

**Table 3:** Morphometric measurements of right and left coracoid process.

Measurements	Indian			Chinese			Myanmarese		
	Right *n* = 12	Left *n* = 12	*P* value	Right *n* = 7	Left *n* = 7	*P* value	Right *n* = 7	Left *n* = 7	*P* value
Length of the coracoid process (mm)	39.15 ± 1.30	39.24 ± 1.57	0.91	43.19 ± 1.44	43.44 ± 1.69	0.69	42.42 ± 0.94	42.51 ± 1.16	0.88
Tip thickness of the coracoid process (mm)	8.70 ± 1.07	8.46 ± 1.07	0.68	11.67 ± 0.72	11.25 ± 0.43	0.11	9.00 ± 0.31	9.16 ± 0.79	0.64
Tip width of the coracoid process (mm)	12.93 ± 1.31	13.12 ± 1.42	0.80	13.59 ± 1.09	13.68 ± 1.13	0.83	13.22 ± 0.57	13.12 ± 0.48	0.73
Base height of the coracoid process (mm)	14.87 ± 0.90	14.71 ± 0.92	0.74	15.63 ± 1.06	16.25 ± 1.53	0.26	15.17 ± 1.23	15.36 ± 1.21	0.77
Base width of the coracoid process (mm)	22.74 ± 0.97	22.97 ± 0.68	0.61	25.37 ± 1.25	25.59 ± 1.76	0.73	23.65 ± 0.78	23.65 ± 0.71	0.22

All values are presented as mean ± SD in mm. *P* value of independent *t-*test was used to identify side differences.

**Table 4:** Distribution of the subjects according to sociodemographic characteristics stratified by ethnicity.

	Ethnicity	*n*	Mean	SD	95% CI for mean	Min	Max	*P* value
Age (years)	Chinese	22	40.54	4.22	38.67–42.42	33	47	0.22
	Malay	11	38.18	6.80	33.60–42.75	27	50	
Height (cm)	Chinese	22	163.95	9.40	159.76–168.13	147	181	0.46
	Malay	11	166.45	8.18	160.95–171.95	155	179	
Weight (kg)	Chinese	22	62.27	15.92	55.21–69.33	36.80	91.50	0.10
	Malay	11	71.56	13.73	62.33–80.78	43.60	94.60	
BMI (kg/m^2^)	Chinese	22	23.17	4.02	21.39–24.95	16.50	30.30	0.13
	Malay	11	25.45	4.13	22.67–28.22	18.10	32.40	

**Table 5:** Morphometric measurements of the length of the coracoid process for both ethnic groups stratified by sex.

Sex	Malay			Chinese			*P* value
	*n*	Mean (mm)	SD (mm)	*n*	Mean (mm)	SD (mm)	
Males	14	39.14	1.16	20	41.80	1.47	0.001
Females	8	34.25	1.28	24	36.20	2.02	0.01
All groups	22	37.36	2.68	44	38.75	3.32	0.09

**Table 6:** Morphometric measurements of the tip thickness of the coracoid process for both ethnic groups stratified by sex.

Sex	Malay			Chinese			*P* value
	*n*	Mean (mm)	SD (mm)	*n*	Mean (mm)	SD (mm)	
Males	14	10.28	0.82	20	11.2	1.05	0.01
Females	8	8.00	0.75	24	9.25	0.94	0.002
All groups	22	9.45	1.37	44	10.13	1.39	0.06

**Table 7:** Morphometric measurements of the base height of the coracoid process for both ethnic groups stratified by sex.

Sex	Malay			Chinese			*P* value
	*n*	Mean (mm)	SD (mm)	*n*	Mean (mm)	SD (mm)	
Males	14	14.92	0.91	20	16.20	0.83	0.001
Females	8	11.75	1.28	24	13.83	1.04	0.001
All groups	22	13.77	1.87	44	14.90	1.52	0.01

**Table 8:** Morphometric measurements of the base thickness of the coracoid process for both ethnic groups stratified by sex.

Sex	Malay			Chinese			*P* value
	*n*	Mean (mm)	SD (mm)	*n*	Mean (mm)	SD (mm)	
Males	14	11.28	1.32	20	11.80	1.80	0.33
Females	8	8.75	0.88	24	9.33	1.71	0.36
All groups	22	10.36	1.70	44	10.45	2.06	0.85

**Table 9:** Morphometric measurements of the coracoid process stratified by gender for both ethnic groups.

Measurements	Malay			Chinese		
	Males *n* = 14	Females *n* = 8	*P* value	Males *n* = 20	Females *n* = 24	*P* value
Length of coracoid process (mm)	39.14 ± 1.16	34.25 ± 1.28	<0.001	41.80 ± 1.47	36.20 ± 2.02	<0.001
Tip thickness of coracoid process (mm)	10.28 ± 0.82	8.00 ± 0.75	<0.001	11.20 ± 1.05	9.25 ± 0.94	<0.001
Base height of coracoid process (mm)	14.92 ± 0.91	11.75 ± 1.28	<0.001	16.20 ± 0.83	13.83 ± 1.04	<0.001
Base thickness of coracoid process (mm)	11.28 ± 1.32	8.75 ± 0.88	<0.001	11.80 ± 1.60	9.33 ± 1.71	<0.001

**Table 10:** Morphometric measurements of the right and left coracoid process for both ethnic groups.

Measurements	Malay			Chinese		
	Right *n* = 11	Left *n* = 11	*P* value	Right *n* = 22	Left *n* = 22	*P* value
Length of coracoid process (mm)	37.40 ± 2.46	37.36 ± 3.00	0.92	38.68 ± 3.51	38.81 ± 3.21	0.89
Tip thickness of coracoid process (mm)	9.45 ± 1.29	9.30 ± 1.50	0.93	10.27 ± 1.35	10.00 ± 1.44	0.52
Base height of coracoid process (mm)	13.81 ± 1.60	13.72 ± 2.19	0.91	15.09 ± 1.50	14.72 ± 1.54	0.43
Base thickness of coracoid process (mm)	10.54 ± 1.69	10.18 ± 1.77	0.62	10.63 ± 1.96	10.27 ± 2.18	0.56
